# Using the CES-D-7 as a Screening Instrument to Detect Major Depression among the Inmate Population

**DOI:** 10.3390/ijerph18031361

**Published:** 2021-02-02

**Authors:** Joel Juarros-Basterretxea, Paula Escoda-Menéndez, Manuel Vilariño, Francisco Javier Rodríguez-Díaz, Juan Herrero

**Affiliations:** 1 Escuela de Psicología y Fiolosofía, Universidad de Tarapacá, Arica 1010069, Chile; 2 Psychology Department, University of Oviedo, 33003 Oviedo, Spain; paula.escodam95@gmail.com (P.E.-M.); gallego@uniovi.es (F.J.R.-D.); olaizola@uniovi.es (J.H.); 3 Political Science and Sociology Department, University of Santiago de Compostela, 15782 Santiago de Compostela, Spain; manuel.vilarino@usc.es

**Keywords:** major depression, prisons, mental health services, screening instruments, CES-D-7

## Abstract

Major depression is one of the most prevalent mental health problems in the penitentiary context and has been related to different undesirable outcomes. The aim of the current research was to evaluate the utility of screening tools for major depression brief assessment in the jail context. We interviewed 203 male inmates and complimented the MCMI-III, the SCL-90-R, and the CES-D-7 self-informed scales. Major depression syndrome and disorder were determined based on MCMI-III criteria and the capability of SCL-90-R and CES-D-7 to identify true positives and true negatives when tested. SCL-90-R and CES-D-7 showed good sensitivity for major depression syndrome and disorder. The specificity of SCL-90-R was poor in all cases, but CES-D-7 showed good specificity depending on the cut-off score. Rigorous interviews are needed for better evaluation of major depression in jails, but screening tools like CES-D-7 are useful for rapid assessment considering the work overload of penitentiary psychologists.

## 1. Introduction

In the last decade more than ever, mental health has become a central social and academic concern. As the historical stigma of mental health problems diminishes, mental health problems have become the core of human health; with this increasing visibility, acceptance, and importance, the necessity for answers has emerged in several fronts [1,2,3]. The growing focus on mental health is fundamentally based on the high prevalence observed around the world, and depression is one of the most prevalent in contemporary societies. Depression affects around 300 million people (4.4% of the world’s population) and is considered the principal risk factor of disability and suicide [4].

These rates are higher in specific populations such as prison inmates [5]. As recent literature has shown, the prevalence of depression in prison is higher than in community samples [6,7,8,9,10,11,12], and despite levels of major depression being slightly lower than psychiatric patients, clinically relevant values have been identified among inmates [13]. Furthermore, not only is depression the most prevalent mental disorder in the penitentiary centers [5,14,15], but major depression is also the most common depressive disorder [5,16,17,18].

Scientific literature identifies depression as a correlating factor and predictor of diverse undesirable outcomes (i.e., suicide, aggressive offenses) [19,20] of paramount importance during incarceration and thus the rehabilitation of inmates. This is particularly important because of the comorbidity of depression with loss of psychological well-being and suicide rates [21,22], especially among prison inmates. Recent research has shown how high suicide risk rates decrease as major depression decreases [19], depicting the contribution of major depression to suicide risk in prison population. This leads to the necessity of accurate assessment of potential cases of major depression for mental health care and suicide prevention in prison context.

The reviewed literature suggests the need to monitor mental health of inmates, and especially major depression evaluations should be routinely carried out. This would allow for the identification of potential cases with a likelihood of other undesirable outcomes such as violent behavior, interpersonal problems, or, in more extreme cases, suicide attempts. However, these initiatives often require personal and material resources and time not always available to mental health professionals [23]. Thus, problems properly assessing inmates’ varying reasons are common and sometimes lie beyond the control of mental health professionals including the professional/inmate ratio [24], which is a crucial limitation for conducting diagnostic interviews and interventions [25].

Unfortunately, the alternatives to the diagnostic interview are based primarily on the application of clinical self-reports such as the Millon Clinical Multiaxial Inventory (MCMI) or the Symptom Checlist-90-Revised (SCL-90-R). The MCMI is designed for measuring different clinical patterns including specific psychiatric disorders. Specifically, it has shown that MCMI’s major depression subscale is concordant with the major depression diagnosis established in clinical interview [26]. Alternatively, the SCL-90-R is also designed to assess different psychological problems and symptoms focusing on the last week and includes depressive symptomatology. This instrument has shown a good measure in different context and it is considered useful for screening [27]. Nevertheless, the utility of this kind of instrument for specific disorders assessment (e.g., major depression) is limited because they consume a considerable amount of time, which can result in a limitation in practice.

At this point, research on brief and reliable screening instruments measuring major depression is a promising area of study with obvious practical implications for prison mental health professionals. These instruments would not only serve to monitor key aspects of the mental health of prisoners during their imprisonment [28,29] but also at the time of entry into prison.

In the present study, we propose the use of a very brief measure of depressive symptomatology (CES-D-7) [30] that comes from one of the instruments for the measurement of depressive symptomatology most widely used by researchers (CES-D). The Center for Epidemiological Studies–Depression scale (CES-D) [31] was originally developed for the study of depressive symptoms in the general population. While the CES-D was not originally design for the evaluation of depressive disorders according to psychiatric criteria, it provides useful information about the presence of depressed mood, feelings of guilt and worthlessness, feelings of helplessness and hopelessness, psychomotor retardation, and somatic complaints, which constitute dimensions of depression. In fact, the CES-D has shown to be an accurate instrument for major depression screening [32,33].

To this end, the present study aimed to analyze the capability of the CES-D-7 for screening major depression in penitentiary accordingly to the MCMI criteria. In order to obtain additional references, the capability of the widely used SCL-90-R’s depression was also estimated in comparison to CES-D-7 considering that both instruments evaluate last-week symptomatology.

## 2. Materials and Methods

### 2.1. Sample

Participants included 203 male inmates in Penitentiary Center of Villabona (Asturias, Spain), 19–66 years old (*M* = 36.73, *SD* = 9.90). Participants belonged to three groups according to their crimes: 44.3% (*n* = 90) for crimes related to intimate partner violence (IPV), 34.5% (*n* = 70) for violent crimes different from IPV (e.g., aggression), and 21.2% (*n* = 43) for nonviolent crimes (e.g., white-collar crimes). Regarding the length of time in prison, 20.5% (*n* = 41) were in prison for less than a year, 37.5% (*n* = 75) about a year, 16.5% (*n* = 33) around two years, and 25.5% (*n* = 51) three or more years. Finally, according to mental health reports available to inmates upon entry into prison, 62.5% (*n* = 125) of participants did not have an official record of any previous mental health problem, 16.5% (*n* = 33) had an official record of diagnosed depression, and 21% (*n* = 42) grouped participants with diagnosed mental health problems other than depression.

### 2.2. Procedure

Researchers approached governmental and penitentiary authorities to explain objectives and obtain permission to perform the study in prison. After obtaining official permission, researchers contacted participants from a provided list of the inmates and then asked them to participate voluntarily in the study. Of all the inmates asked to participate, 98% did. Once the inmates accepted participation, they signed an informed consent and completed a set of self-reported questionnaires. In the present study, only questionnaires pertinent to the aims of the study are considered.

### 2.3. Instruments

Millon Clinical Multiaxial Inventory-III’s (MCMI-III) [34]: The MCMI-III major depression subscale was used, which consists of 17 true-false items (0 = false, 1 = true) (*α* = 0.88; *M* = 5.74, *SD* = 5.52). In MCMI-III, the transformation of direct scores on prevalence scores is used to establish cut-off scores. For the major depression subscale, prevalence ≥ 75 indicates presence of major depression syndrome (MDS) and more prominent manifestation of major depression disorder (MDD) is indicated by prevalence ≥85.

Symptom Checklist-90-R (SCL-90-R) [35]: The SCL-90-R depression subscale was used to measure depressive symptomatology. This subscale consists of 13 items (*α* = 0.92; *M* = 20.69, *SD* = 11.23) measuring the extent to which respondents were concerned about a range of symptoms during the last week. Category responses ranged from 0 (not at all) to 4 (extremely).

Brief Center for Epidemiological Studies-Depression Scale (*CES-D-7*) [30,31]: The CES-D-7 consists of seven items (*α* = 0.82; *M* = 9.70, *SD* = 5.19) that measure frequency of each depressive symptom in the last week. Category responses scale from 0 (never or rarely, less than one day) to 3 (most of the time or all the time, 5–7 days).

### 2.4. Data Analysis

We first estimated the prevalence of major depression syndrome and depression disorder cases by using the MCMI-III major depression subscale cut-off scores [34]. Then, the association between MCMI-III major depression scores to type of crime, time in prison and past history of mental health problems was analyzed using chi-square statistic and phi statistic to estimate the degree of association. At this stage, analysis of variance (ANOVA) was performed to search for differences across groups in major depression direct scores and Hedges *g* was computed to estimate the effect size [36].

Secondly, a receiving operator characteristic (ROC) curve analysis was used to assess the CES-D-7 and SCL-90-R depression subscale performance to detect potential cases of MDS and MDD as provided by the MCMI-III. The accuracy of each instrument was tested by the area under the curve (AUC). AUC values between 0.7 and 0.8 indicate acceptable discrimination, values between 0.8 and 0.9 indicate excellent discrimination and values above 0.9 indicate outstanding discrimination [37]. The effect size is also presented as odds ratio (OR) [38]. The statistically optimal cut-off scores of both instruments are proposed based on sensitivity (≥0.80) and specificity (≥0.80). Nevertheless, given higher relevance of sensitivity to reach the goal of detecting as much as true positives as possible [39], specifically in the prison context, alternative functionally optimal cut-off scores were also proposed based on the higher relevance of the sensitivity (≥0.90) assuming higher rate of false positives (specificity ≥0.50 to avoid the overabundance of false positives). Due to the minor consideration of specificity, functionally optimal cut-off scores are conditional to screening.

## 3. Results

### 3.1. MCMI-III’s Major Depression, Type of Crime, Years in Prison and Antecedents of Mental Health

Following MCMI-III’s criteria, of all study participants, 23 (11.3%) self-reported major depression scores of PREV ≥75 (MDS). Of those 23 inmates, 12 (5.9% of all participants) self-reported major depression scores of PREV ≥85 (MDD).

MDS and MDD were not significantly associated to type of crime (χ^2^_(2)_ = 0.248, *ns.*, φ = 0.035 and χ^2^_(2)_ = 0.334, *ns.*, φ = 0.041 respectively) nor years in prison (χ^2^_(3)_ = 2.259, *ns.*, φ = 0.106 and χ^2^_(3)_ = 3.175, *ns.*, φ = 0.126 respectively). In addition, major depression direct scores were similar across type of crime groups (F_(2, 200)_ = 0.466, *ns.*) and among years in prison groups (F_(3, 196)_ = 0.930, *ns.*).

As far as direct scores in depression are concerned, MDS was significantly associated with antecedents of mental health problems (χ^2^_(2)_ = 6.069, *p* ≤ 0.05, φ = 0.174), but MDD was not (χ^2^_(2)_ = 2.626, *ns.*, φ = 0.115). Analyzing major depression as a continuum revealed significant differences among mental health antecedents groups (*F*_(2, 197)_ = 12.017, *p* ≤ 0.001). Specifically, participants without antecedents of mental health (*M* = 4.30, *SD* = 4.91) show significantly lower levels of major depression than participants with depression antecedents (*M* = 8.70, *SD* = 5.52; *p* ≤ 0.001, *g* = 0.87) as well as those with varied antecedents different from depression (*M* = 7.36, *SD* = 5.90; *p* ≤ 0.01, *g* = 0.59). No significant differences were found among participants with depression antecedents and those with antecedents different from depression (*ns.*, *g* = 0.23).

Based on these previous results, participants with depression antecedents and antecedents different from depression were grouped into “inmates with antecedents of mental health problems.” The remaining participants were grouped into ‘inmates without antecedents of mental health’. Chi-square analysis was carried out to estimate association between groups and MDS and MDS. The relative risk was also estimated. As in the first analysis, MDS (χ^2^_(2)_ = 6.056, *p* ≤ 0.01, φ = 0.174) but not MDD (χ^2^_(2)_ = 0.851, *ns.*, φ = 0.065), was significantly associated with antecedents of mental health problems. Specifically, the relative risk of having major depression syndrome was 2.593 times higher in inmates with antecedents of mental health problems than in inmates without antecedents of mental health problems.

### 3.2. CES-D-7 and of SCL-90-R’s Depression Subscale Sensitivity for Screening Major Depression Syndrome and Disorder

Finally, ROC curve analyses were used to assess CES-D-7 and SCL-90-R depression subscale performance to detect potential cases of MDS and MDD as provided by the MCMI-III. Table 1 displays the results: CES-D-7 showed good accuracy in detecting MDS (AUC = 0.819) as well as MDD (AUC = 0.823). Regarding SCL-90-R’s depression subscale, accuracy to detect MDS (AUC = 0.786) and MDD (AUC = 0.771) was fair. The effect sizes were large in all cases.

The functionally and statistically optimal cut-off scores of CES-D-7 and SCL-90-R depressive symptomatology subscale are presented in Table 2. A cut-off score of 21 was the statistically optimal score for CES-D-7 and the most appropriate for research purposes. Nevertheless, cut-off score of 16 was functionally optimal for screening in the penitentiary context; as shown, using a 16 cut-off score a high capability to detect potential presence of true MDS or MDD (90% of true positives well classified), prioritizing the intervention in the maximum number of those cases, albeit assuming higher number false positives (slightly higher than desired 50%). More conflictive results were obtained for SCL-90-R: low specificity is observed through different cut-off scores (33.5–56.7), impeding proposal of a statistically optimal cut-off score (sensitivity and specificity ≥0.80). Regarding functionally optimal cut-off scores, SLC-90-R depressive symptomatology scale show the same sensibility of CES-D-7 for MDS and MDD, but it showed lower specificity in both cases and was far from the desired 50%.

## 4. Discussion

Using data from 203 imprisoned men from the Penitentiary Center of Villabona (Asturias, Spain), the current study aimed to analyze the ability of an instrument created to evaluate last-week depressive symptomatology for the screening of major depression in penitentiary context: CES-D-7.

### 4.1. Major Depression, Type of Crime, Years in Prison and Antecedents of Mental Health

Following standards proposed by [34], 12.3% of participants met criteria of major depression syndrome, and 5.9% met criteria of major depression disorder. The prevalence of major depression detected using the self-reported MCMI-III scores is consistent with results obtained in previous research; for example, a recent review [10] pointed out that around 10% (95% CI 9, 12) of adult male prisoners show major depression [14,16], and [7] found 13.8% (95% CI 9.7, 18.4) prevalence of major depression for male inmates of nonadmission samples. No association was observed between major depression (syndrome or disorder) and type of crime (IPV, violent crimes different from IPV, and nonviolent crimes) nor years in prison (<1 and ≥3 years) consistently with previous research [17]. These results emphasized the necessity to assess major depression in all type of cases and different stages of imprisonment to guarantee adequate monitoring of evolving major depression, for example, in the form of screening [25]. Current diagnoses of major depression syndrome and disorder were significantly associated with previous diagnoses of depression as well as other mental health problems pointing out the relevance of antecedents as potential indicator of current psychological problems [28].

### 4.2. CES-D-7′s Sensitivity for Screening Major Depression Syndrome and Disorder

The results obtained in the current study indicate that CES-D-7 can be used as screening tool for preliminary assessment of potential major depression cases. The CES-D-7 showed good accuracy in detecting major depression syndrome and disorder being able to correctly classify more than the 80% of the cases. Following a statistical criterion for cut-off score selection, it has shown that the 21-cut-off score had good sensitivity and specificity (≥0.80) for major depression syndrome, and excellent sensitivity (≥0.90) and acceptable specificity (≥0.70) for major depression disorder. Additionally, the CES-D-7 has shown good properties accordingly to functional or utility criteria: Selecting a score of 16 as cut-off allow to correctly classify almost all the real cases (sensitivity ≥0.90) of major depression syndrome and disorder. Despite the higher sensitivity is get at expense of specificity, the 16-cut-off score allow to maintain the false positives near to the 50%.

Second, the CES-D-7 also shown better performance than the SCL-90-R depression subscale, which correctly classified less than the 80% of the cases. Following the same criteria described above, the no statistically optimal cut-off score can be proposed for the SCL-90-R depressive symptomatology because of its generalized poor specificity (≤0.60). Regarding functionally optimal cut-off score, the results shown that a 1.19 score was the best option and it shown the same capability for correctly classifying true major depression syndrome and disorder cases as the CES-D-7 16-cut-off score. Nevertheless, the specificity was extremely poor and less than the 40% of true negatives were well classified.

The results obtained in the current research have important practical implications. Without denying the utility and validity of the SCL-R-90′s depressive symptomatology subscale, the CES-D-7 is an easy to use, briefer, reliable and more accurate instrument for major depression with an important advantage for its potential not only in research but also by the prison-based psychologists which usually complain on the complexity of evaluations in a resource and time limited context like penitentiary centers [23]. The mere shorter nature of the CES-D-7 not only facilitate its use in research context, but also allow to penitentiary psychologist to make more rapid screenings of major depression without lose in the sensitivity and the lower number of false positives also helps to avoiding unnecessary overabundance of clinical interviews after screening [40]. Furthermore, the CES-D-7 also showed an important advantage for its potential differential use depending on the proposal. While the SCL-90-R depressive symptomatology scale’s specificity was poor in all the cases, different cut-off scores can be used with CES-D-7 depending of the main objective of the evaluator; for example, a cut-off score of 21 when using CES-D-7 can be more appropriate for major depression general research because of its good sensitivity and specificity (≥0.80). Alternatively, a higher sensitivity (≥0.90) can be obtained accordingly to the main interest of prison psychologist of avoiding undesirable outcomes related to major depression (e.g., suicide). In this regard, a cut-off score of 16 is a better option in order to detect the maximum number of possible major depression real cases. Despite the better sensitivity is at expense of specificity, the overestimation of positives (false positives) is an assumable error, but the health professionals cannot afford underestimating true positives or overestimating false negatives. Thus, this instrument allows psychologists to prioritize some cases (not implying to ignore others) for a deeper evaluation and to make more precise and prompter identification of major depression cases and design of appropriate interventions [41].

It is important to note we do not advocate generalized use of self-reported measures of major depression (nor other mental health problems) diagnosis. Self-reported approaches tend to overestimate prevalence of mental syndromes and disorders, and particularly screening tools have been strongly criticized due to susceptibility to false positives [10]. Nevertheless, the usefulness of self-reported instruments (e.g., MCMI-III) and screening tools (e.g., CES-D-7) is undeniable in the research context as well as the penitentiary context [42]. As [10] discussed, research should go beyond merely prevalence reports; generating valid and practical knowledge is pivotal when considering the inherent risk of prison for inmates with mental health problems, like suicide, self-harm, violence, and victimization.

The current research extends the literature on major depression screening in the penitentiary contexts proposing a brief screening tool basing on larger and empirically supported measure instruments. The demonstrated utility of the CES-D-7 for the major depression detection among inmates permit to offer a compromise solution to work-overloaded penitentiary psychologists. Though it is not the solution for the real problem which requires more human resources (more psychologists, etc.) to attend to a large number of inmates, it allows psychologists to prioritize the potentially riskier cases in-deep evaluation in a clinical interview to face the current status of mental health care in prisons. Nevertheless, the research also has potential limitations; notwithstanding the importance to extending the knowledge of the particular community of inmates’ reality and needed assessment tools, the sample was not representative and any generalization should be made cautiously. Furthermore, the research does not attend to all the diversity, and future research should analyze the utility of the CES-D-7 in women inmates also, not included in the current research due to the lack of permissions. Regarding the previous information, the research team had available the mental health reports available to inmates upon entry into prison permitting to distinguish participants without an official record of any previous mental health problem, participants with an official record of diagnosed depression, and participants with diagnosed mental health problems other than depression. Nevertheless, the information was scarce and did not allow further analysis.

## 5. Conclusions

The penitentiary context has particular conditions like lack of material and human and time resources [5,23,24,43], which makes it difficult to always carry out appropriate assessments and, consequently, to facilitate the proper intervention [25]. Considering the limitations for an efficient evaluations, brief screening tools like CES-D-7 provide health professionals with an easy to use and short first assessment in order to detect the potential cases of major depression is very useful. The CES-D-7 can be used as a reliable approximation of the scores of diagnostic scales that evaluate depression, specifically the MCMI–III’s Major Depression subscale. This instrument permits rapid evaluation of potential presence of major depression syndrome/disorder and establishes deeper assessment priorities with special emphasis on suicide prevention, essential in any setting with offenders with mental health problems [44]. Due to its characteristics—a measure instrument with high sensitivity, brief and easy to understand and use—it can be used for primary evaluation and priority assignment to different cases for deeper evaluation and intervention implementation. It can also be used for efficient monitoring of inmates to anticipate potential undesirable consequences derived from major depression as suicide attempts. Summing up, this instrument gives penitentiary psychologists an opportunity to briefly assess inmates’ depressive symptomatology and to better account for the riskier cases in order to take action.

## Figures and Tables

**Table 1:** The area under the curve of CES-D-7 and SCL-90-R’s depression subscale.

	AUC	*SE*	Correctly Classified	Odds Ratio
MDS-MCMI-III				
CES-D-7	0.819 ***	0.050	81.9%	10.363
SCL-90-R-depression	0.786 ***	0.050	78.6%	7.636
MDD-MCMI-III				
CES-D-7	0.823 ***	0.075	82.3%	10.778
SCL-90-R-depression	0.771 ***	0.071	77.1%	6.708

*Note:* MDS = Major depression syndrome; MDD = Major depression disorder; *** *p* ≤ 0.001.

**Table 2:** Comparison of possible cut-off scores of CES-D-7 and SCL-90-R’s depression subscale.

	Major Depression			
	Syndrome		Disorder	
	Sens (%)	Spec (%)	Sens (%)	Spec (%)
CES-D-7				
7	100	0	100	0
8	95.7	2.2	91.7	2.1
9	95.7	3.9	91.7	3.7
10	95.7	6.7	91.7	6.3
11	95.7	15	91.7	14.1
12	95.7	20	91.7	18.8
13	95.7	27.8	91.7	26.2
14	95.7	34.4	91.7	32.5
15	95.7	42.8	91.7	40.3
**16**	**95.7**	**47.8**	**91.7**	**45**
17	82.6	53.9	91.7	52.4
18	82.6	63.9	91.7	61.8
19	82.6	66.7	91.7	64.4
20	82.6	75	91.7	72.3
*21*	*82.6*	*80*	*91.7*	*77*
SCL-90-R depression				
0.04	100	4.4	100	4.2
0.12	100	8.9	100	8.4
0.20	100	10	100	9.4
0.27	100	11.7	100	11
0.35	100	13.3	100	12.6
0.47	100	16.1	100	15.2
0.58	100	19.4	100	18.3
0.66	100	20.6	100	19.4
0.77	100	21.7	100	20.4
0.93	100	22.2	100	20.9
1.04	100	23.3	100	22
1.12	100	27.2	100	25.7
**1.19**	**95.7**	**35.6**	**91.7**	**33.5**
1.26	87	38.9	83.3	37.2
1.34	87	40.6	83.4	38.7
1.42	82.6	46.1	83.3	44.5
1.50	82.6	50.6	83.3	48.7
1.57	82.6	53.9	83.3	51.8
1.65	78.3	56.7	75	54.5

*Note.* Sens = Sensitivity; Spec = Specificity; Statistically cut-off scores are in *italics* and functionally optimal cut-off scores are in **bold**. Only those cut-off scores with sensitivity higher than 0.75 were considered.

## Data Availability

The data presented in this study are available on request from the corresponding author. The data are not publicly available due to privacy and ethical restrictions.
